# The Burden of Non-Infectious Organ-Specific Immunopathology in Pediatric Common Variable Immunodeficiency

**DOI:** 10.3390/ijms26062653

**Published:** 2025-03-15

**Authors:** Aleksandra Szczawińska-Popłonyk, Julia Bekalarska, Kacper Jęch, Nadia Knobloch, Oliwia Łukasik, Aleksandra Ossowska, Jędrzej Ruducha, Zuzanna Wysocka

**Affiliations:** 1 Department of Pediatric Pneumonology, Allergy and Clinical Immunology, Institute of Pediatrics, Poznan University of Medical Sciences, 61-701 Poznań, Poland; 2 Student Scientific Society, Poznan University of Medical Sciences, 61-701 Poznań, Poland; j.bekalarska@gmail.com (J.B.); jeca.to.jeca.37@gmail.com (K.J.); nadia.knobloch@gmail.com (N.K.); oliwialukasik11@gmail.com (O.Ł.); ao.ossowska@gmail.com (A.O.); jedrekruducha@gmail.com (J.R.); zuzannawysocka11@gmail.com (Z.W.)

**Keywords:** autoimmunity, common variable immunodeficiency, enteropathy, granulomatous lymphocytic interstitial lung disease, immune dysregulation, nodular regenerative hyperplasia

## Abstract

The pediatric common variable immunodeficiency (CVID) is the most frequent symptomatic antibody production defect characterized by infectious and non-infectious autoimmune, inflammatory, and lymphoproliferative complications. The background for CVID-related organ-specific immunopathology is associated with immune dysregulation and immunophenotypic biomarkers with expansion of CD21low B cells, and dysfunctional memory B cell, follicular T cell, and regulatory T cell compartments. The ever-increasing progress in immunogenetics shows the heterogeneity of genetic background for CVID related to the complexity of clinical phenotypes. Multiple systemic modulatory pathways are determined by variants in such genes as *TACI* or *TNFRSF13B* gene encoding for BAFF-R, *CTLA*-4, *LRBA*, *NFKB1* and *NFKB2*, and *PIK3CD* or *PIK3R1*. The organ-specific immunopathology encompasses a spectrum of disorders associated with immune dysregulation, such as granulomatous interstitial lung disease, hepatocellular nodular regenerative hyperplasia, enteropathy, neuropathy, endocrinopathies, and dermatoses. This review is aimed to define and delineate the organ-specific immunopathology in pediatric CVID. It is also conducted to gather data facilitating a better understanding of complex and heterogeneous immunophenotypes in the context of immune dysregulation mechanisms and genetic background determining manifestations of the disease and implicating personalized targeted therapies with biological agents.

## 1. Introduction

Common variable immunodeficiency (CVID) is the most prevalent symptomatic inborn error of immunity (IEI) characterized by a deficiency of antibody biosynthesis. The diagnostic criteria defined by the International Consensus Document (ICON) [1] and the European Society for Immunodeficiency (ESID) Registry [2] characterize CVID by low serum IgG levels, accompanied by decreased IgM and/or IgA, as well as impaired specific antibody response to protein and polysaccharide vaccines. Exclusion of other specific causes of hypogammaglobulinemia, such as age-related immaturity of immunoglobulin production, infections, disorders of protein losses, protein–energy deficits of malnutrition, malignancies, as well as immunosuppressive therapies [3], is also mandatory to document the primary nature of antibody deficiency. Immunodiagnostic criteria for CVID in children include antibody deficiency interpreted regarding age-matched reference values, along with low switched memory B cell numbers, below 70% of age-related normal values, concurrently without evidence of profound T cell deficiency, low CD4 T helper cell counts, low relative numbers of CD4 T cells to the child’s age, and absent T cell proliferation [2]. Whereas pediatric CVID is predominantly characterized clinically by recurrent respiratory and gastrointestinal infections [4,5,6,7], immune dysregulation-related, non-infectious organ-specific immunopathology frequently determines the clinical phenotype in pediatric CVID patients. Autoimmune, allergic, inflammatory, lymphoproliferative, and malignant disorders are associated with multiorgan hematologic, pulmonary, gastrointestinal, dermatologic, endocrine, cardiac and vascular, bone and joint, as well as neurological symptomatology [7,8,9,10,11,12]. A broad spectrum of molecular immunogenetic pathways is involved in the pathogenesis of CVID, such as, but not limited to, B-cell receptor (BCR) costimulatory B-cell surface proteins, tumor necrosis factor superfamily receptors and ligands, lipid signaling molecules, actin cytoskeleton regulators, transcription factors mediating differentiation and crosstalk, and metabolic processes of glycosylation and mitochondrial pathways [13]. Pediatric CVID patients with the autoimmune and inflammatory phenotype most frequently presented with cytopenias, particularly thrombocytopenia or hemolytic anemia, or both in the form of Evans syndrome, as well as autoimmune neutropenia. Other autoimmune diseases in children affected with CVID were endocrinopathies, such as thyroiditis and diabetes [10,14], inflammatory arthritis [10,15], dermatologic disorders, such as alopecia and vitiligo [10,16], gastrointestinal complications, namely celiac disease, and inflammatory bowel disease [10], as well as systemic lupus erythematosus (SLE) [8]. In these patients, in whom a monogenic background of CVID was identified, variants in genes, such as *cytotoxic T lymphocyte antigen 4* (*CTLA4*), *lipopolysaccharide (LPS)-responsive beige-like anchor protein* (*LRBA*), *BTB domain and CNC homolog 2* (*BACH2*) [17,18], *nuclear factor kappa B subunit 1* (*NF-kB1*) [17,18,19], *signal transducer and activator of transcription 3* (*STAT3*) [18,20], *phosphoinositide 3-kinase* (*PI3K*), *inducible T-cell costimulator* (*ICOS*), *IKAROS family zinc finger 1* (*IKZF1),* or *interferon regulatory factor 2-binding protein 2* (*IRF2BP2*) [19] were demonstrated. Noticeably, a complex and heterogeneous inflammatory, autoimmune, and lymphoproliferative CVID phenotype implicates different modes of inheritance with variable degrees of expressivity and penetrance. Genotype–phenotype correlations in CVID are even more conveyed by a predisposition to the disease, as exemplified by variants in *Transmembrane Activator and CAML Interactor* (*TACI*), aka tumor necrosis factor receptor superfamily member 13B (TNFRSF13B), which may not be causative for CVID but may coexist and interact synergistically with other variants showing deleterious effects. Consequently, the disease symptomatology may be determined by epistatic interactions, i.e., synergistic interplay of two genetic loci that substantially modify the disease severity or result in entirely new phenotypes, as well as by the effect of gene additivity [20]. Noticeably, the epistasis phenomena may be exerted by digenic variants in genes in which products are playing roles in the same physiological pathways, e.g., variants in *TACI*, stimulating a T-cell-independent class-switch recombination (CSR), and *transcription factor 3* (*TCF3* aka *E2A*), playing a role in T-cell-independent and T-cell-dependent immunoglobulin class switching and secretion with a clinical phenotype of immunodeficiency and autoimmunity [21]. It has also been assumed that other variants in genes, such as *NFKB1* and *nucleotide-binding oligomerization domain containing 2* (*NOD2*) [22], *LRBA* and *Nei-like DNA glycosylase 3* (*NEIL3*) [23], and *CTLA4* and *Janus Kinase 3* (*JAK3*) [24], are related to clinical autoimmune and inflammatory epistasis in CVID.

The complex genetic and pathophysiological underpinnings of pediatric CVID determining the heterogeneity of the phenotypic features and complex symptomatology of the disease are challenging for clinicians. This review is therefore aimed to resume and conclude the organ-specific immunopathology in pediatric CVID. It is also conducted to provide data facilitating a better understanding of complex and heterogeneous immunophenotypes in the context of immune dysregulation mechanisms determining manifestations of the disease. Due to the paucity of clinical and experimental studies on immune dysregulation and organ-specific immunopathology in pediatric CVID, this review provides a novel insight into their pathogenesis.

## 2. Immunopathogenetic Background

In search of immunopathogenetic denominators relevant for immune dysregulation in pediatric CVID, a spectrum of biomarkers was analyzed, including circulating immune cells, serum immunoglobulins, regulatory pro- and antiinflammatory cytokines, lipid indicators, as well as immunophenotypes of peripheral blood lymphocyte compartments [8,9,11,12,25]. Attempts have been made to uncover correlations between experimental parameters of the immune response and clinical phenotypes associated with immune dysregulation to precisely stratify CVID patients with an increased risk of consequent organ-specific immunopathology. Ultimately, insight into the immunopathogenetic background and defining high-risk individuals could potentially contribute to targeted treatment approaches and the implementation of novel therapies.

It has been demonstrated that B cell lymphopenia correlated with hematologic, rheumatologic, and gastrointestinal autoimmune and inflammatory disorders in CVID patients and thereby proved to be an indicator of immune dysregulation [8].

The relative abundance of serum IgM levels was also suggested to indicate a high risk of non-infectious complications in CVID. Higher-baseline serum IgM was noted in those patients who developed lymphoma and CVID-associated progressive interstitial lung disease (ILD) and the latter organ-specific immunopathology correlated with B cell hyperplasia and germinal center formation in the lungs. Thereby, increased serum IgM level, reflecting a defective immunoglobulin class-switch recombination (CSR) may be used as a simple and relevant biomarker of immune dysregulation in the lung [25]. Noticeably, polyautoimmunity was also noted in children affected with other forms of IEI paralleled with the hyper-IgM (HIGM) phenotype, e.g., class-switch recombination defects due to genetic variants in the *IKBKG* gene, aka *NEMO*, regulating the activity of nuclear factor kappa B 1 (NFκB1), and subsequent deregulation of activation-induced cytidine deaminase (AID) and uracil DNA glycosylase (UNG) enzymatic activity [26], as well as HIGM manifestation of ataxia-telangiectasia [27,28]. Whereas IgG and IgA deficiency is a hallmark of CVID, increased serum IgM levels reflect impaired B cell differentiation and deregulation of germinal center reaction, and dysfunctional T cell help may be potential immunopathogenetic mechanisms of immune dysregulation.

It has also been suggested that beyond abnormalities of adaptive immunity, activation of acute phase reactions plays a contributory role in systemic activation of immune pathways in CVID. To support this hypothesis, soluble lipopolysaccharide (LPS) binding protein (LBP) and a cell surface antigen CD14, a co-receptor for toll-like receptors (TLR) on macrophages and monocytes activating innate immune responses, were shown to be abundant in CVID individuals with non-infectious manifestations [29,30]. Furthermore, in those patients, elevated levels of sCD14 were correlated with sCD25 on activated T cells [25]. Another candidate biomarker of systemic inflammatory response in CVID is HDL due to its low serum levels, and impaired function was revealed in higher frequencies in patients with CVID-related immune dysregulation [31].

A deregulated serum cytokine milieu and protein mediator environment was demonstrated in individuals with CVID and non-infectious complications, such as inflammatory bowel disease, interstitial lung disease, and chronic liver disease. The patients showed elevation of serum proinflammatory cytokines, interleukin (IL)-1β, IL-6, tumor necrosis factor alpha (TNF-α) [32], and also IL-18, IL-12p40, mediators, such as lymphotoxin alpha, oncostatin M, and vascular epithelial growth factor (VEGF). Moreover, elevated levels of a range of proteins involved in T cell functions were also assessed, including T cell co-stimulating factor TNFRSF5 or CD40, a cellular signal modulating T and B1 cell surface molecule CD5, and CD6, restraining signal transduction upon T cell activation [30]. Another finding demonstrates that patients with non-infectious autoimmune and inflammatory complications have elevated interferon (IFN) signature genes and subsequent expansion of IFN-γ producing innate lymphoid cells, which are regulators of innate immune response [33]. Interestingly, marked cytokine dysregulation was observable in those CVID patients who received subcutaneous or intravenous immunoglobulin replacement therapy (IgRT), implicating a poor preventive immunoregulatory effect on autoimmune and inflammatory phenomena in CVID [32], thereby leaving the space for patient-tailored novel therapeutic approaches.

Numerous abnormalities within the peripheral blood lymphocyte compartment resulting in a skewed immune response were demonstrated in CVID associated with autoimmune and inflammatory disorders. Defective differentiation and maturation of B cell subsets, with B cell lymphopenia, low switched memory CD19^+^CD27^+^IgD^−^ B cells, and expansion of immature activated CD19^+^CD38^lo^CD21^lo^ B cells are an immunopathogenetic feature of CVID. These defects in B cell subpopulations were linked to autoimmune diseases, such as autoimmune hemolytic anemia (AIHA), autoimmune thrombocytopenia (ITP), Evans syndrome, vitiligo, and systemic lupus erythematosus (SLE), as well as interstitial lung disease (ILD) in CVID cohort [34,35]. Noticeably, autoreactive active naïve and double-negative 2 (DN2) B cells, demonstrated in SLE, proved to be common among CVID patients with autoimmune features, suggesting a common pathogenesis associated with failure of B cell tolerance and a role in the development of autoimmunity in CVID [36]. The impaired development and functional abnormalities of B cells, encompassing class-switch recombination defects, impaired antibody affinity maturation, and expansion of immature B cell population result from deregulated multiple pathways and T cell–B cell interaction, signaling, and activation processes. While profound T cell deficiency is an exclusion criterion for CVID according to the ESID definition [2], multiple defects in the T cell compartment were reported. They included significantly reduced naïve T helper cell and recent thymic emigrant (RTE) cell counts, expanded CD4^+^CD45RO memory T cell population, along with excessive T CD4^+^ cell activation corresponding with organ-specific autoimmunity, cytopenia, enteropathy, polyclonal lymphoproliferation, and lymphoid malignancy [34,37,38,39]. Variants in several well-known genes, such as *LRBA*, *CTLA*, *BACH2*, *STAT3*, and *IKAROS*, as well as those recently described, such as *guanine nucleotide exchange factor* aka *IRF4BP* (*DEF6*), *Ferm domain containing kindlin* 1 (*FERMT1*), and *interleukin 2 receptor subunit beta* (*IL-2RB* or *CD122*), are associated with disorders in immune regulatory pathways and inflammatory, autoimmune, atopic, and lymphoproliferative phenotypes, and thereby have been categorized as primary immune regulatory disorders (PIRD). The central pathophysiological role in inborn errors of immune dysregulation is related to the defects in numbers and suppressive effector functions of CD4^+^CD25^+^Foxp3^+^ regulatory T (Treg) cells [37,40]. Interestingly, in pediatric CVID patients presenting with autoimmune disorders, such as cytopenia and enteropathy, the transcriptome-wide Treg cell profiling revealed altered gene signatures of Treg cells associated with the downregulation of class I IFN signaling pathways [41]. Beyond CD4^+^CD25^+^Foxp3^+^ Treg cells, a spectrum of regulatory cells involved in immune homeostasis and deregulated in CVID have been reported. Among T cells, it is worth noting CD8^+^CD25^+^Foxp3^+^ Treg cells, CD8^+^CD28^−^Foxp3-Treg cells producing IL-10 and tumor necrosis factor beta (TGF-β), invariant immunoregulatory iNKT CD4^+^CD8^+^ cells, and in the B cell compartment, regulatory B cells with CD19^+^CD24^hi^CD27^+^ immunophenotype and secreting IL-10 [42].

## 3. Organ-Specific Immunopathology

### 3.1. The Respiratory Tract

The pediatric CVID-associated pulmonary immunopathology encompasses two distinct entities, structural airway disease, e.g., bronchiectasis and bronchial wall thickening as well as interstitial or parenchymal lung disease (ILD) [43]. The development of chronic structural airway disease with bronchiectasis and peribronchial thickening has been primarily ascribed to adult patients affected with CVID and etiologically linked with cumulative recurrent and prolonged respiratory tract infections, hence their frequencies occur progressively. Upper airway infections, e.g., rhinosinusitis or chronic otitis media, and also recurrent bronchitis, are the most common risk factors for bronchiectasis at every age, yet in adult CVID, patients are frequently accompanied by asthma and chronic obstructive pulmonary disease as independent predictors [44]. Since respiratory infectious episodes are hallmarks of childhood CVID, bronchiectasis is perceived as a common complication present in as many as 25% of newly diagnosed pediatric patients [45,46]. The development of bronchiectasis in children with an early-onset CVID, suggesting a monogenic disease, e.g., LRBA, CTLA, or PIK3CD defects, implies a deeper insight into its pathogenesis. The mutual genotype–phenotype relationships linked with immune dysregulation and chronic inflammatory response may, in consequence, lead to structural airway damage [47]. Furthermore, CVID patients with bronchiectasis have significantly lower IgA and IgM serum levels, lower total B cell and switched memory B cell numbers, expanded CD21^lo^ immature B cells, as well as fewer T CD4^+^ cells than those without bronchiectasis [47,48]. These complex immunological disturbances may contribute to the increased susceptibility to systemic inflammation and organ-specific immunopathology in the form of bronchiectasis.

Beyond the structural airway pathology, interstitial lung disease (ILD) is an inflammatory non-infectious organ-specific pulmonary complication including interstitial and parenchymal disorders affecting CVID patients. ILD encompasses several clinical, radiological, and histopathological conditions, such as ground-glass opacities, consolidations, and nodules corresponding with follicular lymphocytic hyperplasia and granulomas. The granulomatous lymphocytic interstitial lung disease (GLILD) is primarily ascribed to CVID, yet systemic excessive inflammatory response with granulomatosis was also assessed in other pediatric syndromic IEI disorders, such as hyper-IgE (STAT3-HIES), CDC42 deficiency [49], 22q11.2 deletion [50], and Nijmegen breakage syndrome [51]. Inasmuch both pulmonary and extrapulmonary, liver, spleen, lymph nodes, and skin granulomatous inflammation is not exclusively a feature of CVID, but it is recognized in other IEI categories, such as combined immunodeficiencies, DNA repair defects, phagocytic disorders, and primary atopic and autoinflammatory diseases [52], it may therefore be assumed that GLILD may encompass several distinct pathologies. The assessment of granulomas in CVID for histopathological characteristics show they are small and poorly circumscribed, with few multinucleated giant cells and minimal fibrosis [53]. Because the diagnosis of GLILD is commonly established based on imaging and the lung in children is rarely sampled, the availability of histopathology in childhood CVID-associated GLILD is limited. In those individuals in whom lung biopsies were performed, granulomas were accompanied by lymphoid interstitial pneumonia, lymphoid hyperplasia, follicular bronchitis, and pulmonary fibrosis [54].

While in search of GLILD immunological biomarkers and predictors, multiple phenotypic features of lymphocyte deregulation have been shown, however, the pathogenesis of this condition has not been fully elucidated. It is perceived as an organ-specific immunopathology that is a manifestation of systemic autoimmune, inflammatory, and lymphoproliferative complications, as affected patients frequently manifest splenomegaly, lymphadenopathy, and cytopenia. A severe decrease in switched memory B cells, expansion of CD21^lo^ B cells, and increased serum levels of B cell activating factor (BAFF) are found in progressive GLILD, implicating its role in resistance to apoptosis and inducing B cell hyperplasia and defective B and T cell crosstalk, and activation in *NFKB1* and *PIK3CD* defects were revealed [55]. Among predominant T cell disorders contributing to the breakdown of immune tolerance in GLILD, increased frequency of HLA-DR^+^CD4^+^ and exhausted CD8^+^CD57^+^ central memory T cells were demonstrated [55]. Interstitial lung disease with granulomas was also reported in patients with LRBA and CTLA deficiencies belonging to a category of IEI characterized by T regulatory cell dysfunction and thereby predisposition to immune dysregulation and autoimmunity [56].

Importantly, GLILD is a common complication affecting about 10–15% of CVID patients [57], worsening the prognosis at every age and significantly reducing the life expectancy, and thereby entailing further studies in pediatric CVID on the burden, genotype–phenotype correlations, immunological underpinnings, and disease biomarkers in a search of targeted therapies.

### 3.2. The Gastrointestinal Tract

Gastrointestinal non-infectious inflammatory, autoimmune, and lymphoproliferative complications commonly affect CVID patients and are observable in up to 20% of them. They are characterized by gastric, both small and large intestine, as well as liver disease, which may be accompanied by extragastrointestinal manifestations, such as GLILD, juvenile idiopathic arthritis, systemic lupus erythematosus, vasculitis, thyroiditis, and autoimmune cytopenia [57,58]. The CVID-related intestinal organ-specific immunopathology is associated with a reduced life expectancy and marked individual burden due to chronic diarrhea, malnutrition, and proneness to lymphoma. Chronicity of enteropathy poses the risk of nutritional deficiencies, intestinal protein loss, vitamin deficiency, osteopenia and osteoporosis, malabsorption, and anemia, which is multifactorial due to iron malabsorption, disturbed hematopoiesis, and intestinal blood loss [59].

The histopathology of CVID-related enteropathy is heterogeneous, encompassing a wide range of morphological features [60]. The histological findings in enteric biopsies may include villous atrophy, nodular lymphoid hyperplasia with intra or subepithelial lymphocytosis, prominent apoptosis, granulomas, and crypt distortion, accompanied by absence of plasma cells in the lamina propria [60,61]. This altered bowel histology reveals distinct features of mucosal inflammatory immune mechanisms falling into the category of an inflammatory bowel disease (IBD)-like condition [62]. Of note, a high incidence of infectious etiology due to *Campylobacter*, enteropathogenic *E. coli*, *Giardia lamblia*, or *norovirus* was shown in CVID patients with inflammatory colitis and florid nodular lymphoid hyperplasia manifesting as chronic diarrhea and weight loss, thereby implicating etiological hints [63].

In search of immunogenetic indicators of CVID-related IBD-like colitis, pathogenic variants in several genes linked to immune dysregulation and immunodeficiency, such as *CTLA*, *PIK3CD* associated with activated phosphoinositide 3-kinase delta syndrome 1 (APDS1), *PIK3R1* (APDS2), *TNFRSF13B* (TACI), *NFKB1*, *NFKB2*, and *protein tyrosine phosphatase, non-receptor type 2* (PTPN2) [60].

In the upper part of the gastrointestinal tract, immunopathology of the stomach and duodenum was reported in CVID. In a large proportion of patients, chronic erythematous, follicular, atrophic, or ulcerative gastritis is diagnosed. Of note, in the development of the gastric chronic inflammatory mucosal process, *Helicobacter pylori* is not the only contributor, but rather immune dysregulation also plays a primary role. In adult CVID patients, atrophic gastritis and intestinal metaplasia are associated with an increased incidence of stomach cancers, such as carcinoma and lymphoma [60,64].

An association between celiac disease and CVID has also been hypothesized due to histological findings resembling this condition, with increased numbers of intraepithelial lymphocytes, mononuclear cell infiltration of lamina propria, and villous atrophy in biopsied patients [65,66]. Importantly, several histopathological features may distinguish between celiac disease and a CVID-related celiac-like disorder, which is characterized by lower intraepithelial and often partial lymphocytosis, presence of follicular lymphoid hyperplasia, and strong neutrophil infiltration. In a celiac disease-related HLA profile, HLA-DQ2 and HLA-DQ8 are helpful in distinguishing a true celiac from a celiac-like disease [65]. The histopathological findings of the celiac-like disease most probably reflect an immune dysregulation phenotype with persistent immune activation and inflammation, characterized by soluble IL-2 receptor (sCD25), LPS surface receptor of monocytes and macrophages (sCD14), as well as their low-grade inflammation marker (sCD163) [65].

The liver disease in CVID is particularly common as persistently deranged liver function is observable in as many as 50% of CVID patients [67]. Beyond infections, this organ-specific immunopathology encompasses two important conditions resulting from immune dysregulation, nodular regenerative hyperplasia (NRH), and malignancies. The first disorder is perceived as an intrahepatic vasculopathy, which may occur in many liver diseases, leading to hepatocyte injury and regeneration with subsequent development of nodules compressing sinusoids and veins, or persinusoidal fibrosis resulting in portal hypertension aka port-sinusoidal vascular disease (PSVD). The pathogenesis of NRH and PSVD may be associated with a spectrum of processes, such as thrombosis and vascular obliteration, chronic cytotoxic CD8^+^ T lymphocyte infiltration of the sinusoidal endothelium, microbial dysbiosis and translocation of proinflammatory bacteria and endotoxins to extraintestinal regions, as well as autoimmune hepatitis-like liver injury unique for CVID [68]. Accumulation of endothelial cytotoxic T cells and deregulated intestinal microbiome thereby disturbing the gut-liver axis in CVID may result in liver failure due to severe enteropathy [69,70,71].

The immunophenotype with liver involvement was linked to several monogenic IEI diseases and CVID presentations. Loss-of-function (LOF) variants in *inducible T cell costimulator* (ICOS), *NFKB1*, *NFKB2*, *CTLA-4*, *LRBA*, *adenosine deaminase 2* (ADA2), *IL-21 receptor* (IL21-R), as well as gain-of-function (GOF) variants in *PI3KCD* associated with immune dysregulation clinical phenotypes, including autoimmune cytopenia, inflammatory skin disease, arthritis, lymphoproliferation, and endocrinopathies, were found in patients with liver disease, supporting the hypothesis of the heterogeneity of organ-specific hepatic disorders in monogenic IEI disorders [67].

### 3.3. Central and Peripheral Nervous System

Neurological non-infectious inflammatory, autoimmune, or malignant complications in pediatric common variable immunodeficiency may involve both the central and peripheral nervous system. Neurological manifestations are admittedly less prevalent than in other categories of IEI diseases, such as syndromes associated with immune deficiency, e.g., 22q11.2 deletion syndrome, ataxia-telangiectasia, and other DNA reparation defects, or purine nucleoside phosphorylase (PNP) deficiency, that are characterized by a broad spectrum of structural and functional disorders [72,73,74,75]. Whereas infectious meningoencephalitis is the most frequent etiological mechanism of neurological sequelae in children, the immune dysregulation process plays a pivotal role in their immunopathogenesis, and multiorgan dysfunction, neurotoxicity as a treatment complication, and metabolic disturbances, such as electrolyte or vitamin deficits, may also be important contributory factors [72,73].

The reported neurological symptomatology among patients with CVID diagnosis predominantly includes manifestations of the central nervous system involvement, such as headache, developmental delay, seizures, vertigo, impaired sensory and motor functions, hypoacusis and impaired vision acuity, movement disorders, optic neuritis, and in addition to somatic problems, neuropsychiatric conditions, such as depression, anxiety, and eating disorders [76,77,78]. Of note, CVID patients presenting with neurological complications, in particular autoimmune encephalitis, tend to be a younger age in childhood at the onset of symptoms, as well as higher incidence of autoimmune disorders besides the central or peripheral nervous system, such as interstitial lung disease, inflammatory colitis, and autoimmune cytopenia [79]. Leptomeningeal involvement, macroscopic vasculitis, space-occupying lesions, as well as granulomatous encephalitis were revealed in those patients with cerebral magnetic resonance imaging (MRI) [80]. Intracranial granulomatosis was frequently accompanied by granulomatous disease of the lungs, liver, spleen, lymph nodes, eyes, and skin [81], reflecting a CVID-related predisposition to the lymphoproliferative process due to the mutual multisystemic pathophysiological background.

The peripheral nervous system immunopathology is rare in pediatric CVID, yet individual patients with transverse myelitis, Guillain-Barre syndrome, and myasthenia gravis were reported. In a child affected with CVID, chronic inflammatory demyelinating polyneuropathy was diagnosed, providing another example of an immune dysregulation condition resulting from an autoimmune response to myelin antigens of the peripheral nerves [82].

Special attention should be paid to the molecular underpinnings associated with LRBA deficiency manifesting as CVID and additionally characterized by regulatory T cell dysfunction, thereby resulting in predisposition to immune dysregulation with autoimmunity and autoinflammation. The burden of neurological disorders concerns about 25% of patients with LRBA deficiency, and both inflammatory brain lesions as well as peripheral cervical transverse myelitis were reported in affected children [83,84].

### 3.4. Bones and Joints

Rheumatologic disorders associated with articular and connective tissue diseases in pediatric CVID are perceived as quite common and occur in about 10% of patients [85]. Among children, juvenile idiopathic arthritis (JIA) is the most prevalent manifestation, corresponding with adulthood rheumatoid arthritis (RA), and followed by juvenile spondyloarthritis (JSpA). Importantly, in a proportion of patients, arthritis and other autoimmune complications, such as idiopathic thrombocytopenic purpura (ITP), autoimmune hemolytic anemia, insulin-dependent diabetes mellitus (IDDM), and inflammatory bowel disease, preceded the rheumatologic presentation and were heralding the diagnosis of CVID [85].

An early-onset severe clinical course of JIA, characterized by bone demineralization and multifocal joint erosions with flexion deformity, was reported in children in whom genetic variants in *LRBA* were identified [86,87]. In these patients, JIA was accompanied by immune dysregulation disorders, such as Evans syndrome, and inflammatory bowel disease, with lymphoproliferation presenting as splenomegaly and lymphadenopathy, which was diagnosed as Rosai-Dorfman syndrome in one of the reported children [88]. Dysfunction of LRBA leads to a deregulation of the CTLA-4 pathway and disorders of the T cell compartment, predominantly a Tregopathy, thereby predisposing LRBA deficient patients to immune dysregulation processes.

Spondyloarthritis involves the axial skeleton in the form of sacroiliitis or ankylosing spondylitis and is associated with a spectrum of relevant extra-articular features, such as anterior uveitis, psoriasis, dactylitis, and inflammatory bowel disease. It may be hypothesized that the complex phenotype sharing features with enteropathic arthritis and spondyloarthritis reflects a common pathomechanism of the gut–joint axis and expanded organ-specific immunopathology [89].

### 3.5. The Skin

Beyond infections, such as abscesses, cellulitis, impetigo, and warts, which constitute the most prevalent group of cutaneous manifestations in CVID, a spectrum of non-infectious autoimmune, inflammatory, and lymphoproliferative disorders has also been reported [90]. Importantly, children with skin manifestations are older at the diagnosis age for immunodeficiency, thereby pointing to the diagnostic delay due to the complex clinical phenotype with cutaneous disorders.

A clinically important yet underestimated group of disorders associated with antibody deficiency in children are allergy complications, in particular, atopic dermatitis due to disturbed T cell functions contributing to the development of atopic skin disease [91]. Multiple co-stimulatory and co-inhibitory pathways regulating the T cell receptor activity have been implicated to play a role in the immunopathogenesis of atopic dermatitis. These include, among others, the B7-CD28 subfamily represented by CLTLA-4, which is expressed largely on Treg cells and is showing a co-inhibitory function suppressing the T cell response B7-CD28 subfamily family encompassing PD-1 inhibitory receptor activating Treg cells, as well as the CD28 family, including ICOS expressed on activated Treg cells and interacting with B7-CD28 costimulators [92]. The chronicity and refractory course of atopic dermatitis also led to the hypothesis of the role of autoimmune response to autoantigens and the progressive transition from allergic inflammation to autoimmune processes [93].

Autoimmunity and hyperinflammatory response make CVID patients susceptible to cutaneous complications such as alopecia, vitiligo, psoriasis, and systemic lupus erythematosus (SLE) [94], which may be accompanied by other extracutaneous autoimmune disorders, such as thyroiditis, celiac disease, myositis, membranoproliferative glomerulonephritis, and Evans syndrome [95]. The highest risk of autoimmune, allergic, and inflammatory skin complications is associated with monogenic forms of diseases manifesting as CVID and categorized as immune regulatory disorders, such as LRBA or NFKB2 deficiencies [96].

Granulomatous skin disease affects about one-third of pediatric patients with CVID [97,98] and is most commonly associated with systemic granulomatosis involving the lung, spleen, liver, and lymph nodes. The histopathologic examination of the biopsied lesions demonstrates inflammatory sarcoid-like lesions.

### 3.6. Endocrine Glands

Hormonal dysfunctions may be a part of the broad spectrum of phenotypic features in pediatric CVID, influencing metabolic mechanisms as well as intellectual, physical, and sexual growth and functions. The pleiotropic regulatory effect of peptide and nonpeptide hormones influences immune cell development and functions. Classic thymic hormones, thymulin, thymopoietin, and thymosins as well as other hormones, such as growth hormone, prolactin, oxytocin, and glucocorticosteroids, regulate the thymic microenvironment and thereby are also involved in T cell proliferation, survival, and selection of the TCR repertoire [99]. Thyroid hormones and thyrotropin (TSH) exert their effect on lymphocyte proliferation and activation by genomic and non-genomic mechanisms and involve the development and functions of B, T, and NK cells [100,101]. Endocrine disturbances are frequently observed in children with CVID due to molecular defects, autoimmune reactions, and chronic inflammatory conditions [9,102], posing the vicious circle of endocrinopathy and immunopathology.

The most common autoimmune endocrine disorders in CVID are thyroiditis and type 1 diabetes mellitus (T1DM). Other autoimmune endocrinopathies in children with CVID are hypoparathyroidism, ovarian failure, hypophysitis, adrenal insufficiency, growth hormone deficiency, and hypogonadism [102,103]. Several monogenic defects manifesting as CVID with immune dysregulation are associated with susceptibility to developing autoimmune endocrinopathies, such as early-onset hypothyroidism or T1DM associated with genetic variants in *LRBA* or *CTLA* [103]. A relevant example of a complex immune deficiency co-occurring with endocrinopathy is a variant in *NFKB2* (p52LOF/IκBδ GOF). The phenotype encompasses an anterior pituitary gland involvement, causing adrenocorticotropic hormone (ACTH) and growth hormone deficiencies and a predominantly B cell dysfunction (DAVID syndrome), which poses the risk for other autoimmune disorders [103,104,105]. Susceptibility to T1DM in CVID has also been reported [106], and in a patient with a monogenic form of the disease, bearing a variant in the *TNFRSF13C* gene encoding for BAFF receptor (BAFFR), it was accompanied by severe immune dysregulation with ITP and peripheral polyneuropathy [107]. Short stature in children with CVID may show genetic and molecular underpinnings, such as variants in *PIK3R1*, resulting in activated PI3K δ syndrome 2 (APDS2), which may be associated with features of SHORT syndrome characterized by short stature, joint hyperextensibility, ocular depression, Rieger anomaly, and delayed tooth eruption [108,109]. However, the attention of pediatricians and immunologists should be drawn to a wide spectrum of inborn errors of immunity which exert their effect on the growth of affected children, and beyond the genetic and molecular background, this influence is multifactorial, encompassing multiple mechanisms, and showing a causal relationship with growth retardation. These include polyautoimmunity, recurrent infections, malabsorption, reduced caloric intake, and catabolic processes [110].

### 3.7. The Cardiovascular System

The cardiovascular involvement in pediatric CVID is rarely reported and sparse data on autoimmune and inflammatory disorders of the heart and vessels are available. Acute pericarditis, which was perceived as a peculiar manifestation of CVID, was diagnosed in 1,5% of the affected patients [111]. Another cardiac condition implicating an autoimmune background due to CVID-related immune dysregulation is giant cell myocarditis. This rare disease is associated with a high rate of fatality due to progressive cardiogenic shock, and in the two reported CVID patients, heart transplantation was a life-saving procedure [112,113].

Takayasu arteritis is a chronic vasculitis involving the aorta and its branches, manifesting as hypertension, increased inflammatory markers, and constitutional symptoms, such as cerebrovascular episodes, local and systemic inflammation, end-organ ischemia, weight loss, and weakness [114,115,116]. The childhood onset of CVID and the developing Takayasu arteritis also suggest a common immune dysregulation pathophysiology, in particular, due to an immunogenetic background of LRBA deficiency [114].

For CVID patients in their transition ages from childhood to adulthood, cardiometabolic disorders may potentially play a contributory role in the pathogenesis of the autoimmune and inflammatory response. Impaired lipid metabolism with elevated serum low-density lipoprotein (LDL) and endothelial cell dysfunction may result in oxidative stress and inappropriate vascular reactivity [117]. Disturbed gut microbiota is linked to a dysregulated lipid profile with triglyceride (TG) and very low-density lipoprotein (VLDL) levels, posing an altered metabolic syndrome strongly associated with LPS, a marker of leaky gut in CVID [118]. Intestinal dysbiosis is also associated with an altered plasma fatty acid (FA) profile and reduced omega 6 polyunsaturated FA, and thereby may predispose patients to lower IgG levels and systemic inflammation, escalating a pathogenic loop of inflammation and metabolic disturbances [119].

### 3.8. Challenges and Future Perspectives

From the era of the clinical and immunological diagnosis of the predominantly infectious phenotype in pediatric CVID, we moved to the era of diagnostic and therapeutic challenges of complex immunophenotypes at the interface of immunodeficiency and immune dysregulation. The immunophenotypes related to molecular background and organ-specific immunopathology [120,121,122,123,124,125,126,127,128,129,130,131,132] are summarized in Table 1. Noticeably, the mutual relationship of infection and immune dysregulation as well as an additive effect of infections, such as *SARS-CoV2*, *herpes viruses*, such as *cytomegalovirus* and *Epstein–Barr virus*, as well as intestinal *norovirus* or respiratory *rhinovirus* and *adenovirus,* on pathophysiology of organ-specific immunopathology should be considered [133,134,135]. Beyond infectious complications that are common clinical manifestations in children with CVID, autoimmune, inflammatory, granulomatous, and lymphoproliferative disorders emerged as an important target for therapeutic interventions. Since the immunogenetic landscape in CVID is complex and encompasses monogenic, digenic, polygenic, as well as epigenetic and environmental underpinnings, the molecular definitive diagnosis is available in a limited proportion of patients [136]. The low rate of genetic diagnoses in individuals with CVID is a challenge for the future as they are fundamental to the therapeutic and prognostic approaches.

The priorities for the precision therapies are organ-specific immune-driven pathologies, such as pulmonary GLILD, NRH in the liver, enteropathy, endocrine disorders, and lymphoproliferative disease of the spleen and lymph nodes. Along with drugs of well-established positions in the therapy of CVID complications, such as the mammalian target of rapamycin (mTOR) signaling inhibitor sirolimus for GLILD, novel immunosuppressive and immunomodulating agents promote and facilitate more effective treatment strategies to protect patients from multisystemic immune dysregulation and organ failure. For example, in GLILD, the anti-CD20 monoclonal antibody rituximab is routinely used, but the anti-BAFF antibody belimumab could be an alternative or a supplement to rituximab in B cell-driven disease [137,138]. Liver NRH is a severe complication of CVID with no established therapeutic options and liver transplantation required in an end-stage case of the disease [139]. For CVID-related enteropathy, anti-TNF agents, such as a receptor fusion protein, etanercept, anti-TNF monoclonal antibodies, infliximab, and adalimumab require further evaluation. Vedolizumab, the integrin antibody, inhibits a T cell migration to the gut, and ustekinumab, an IL-12 and IL-23 inhibitor, as well as guselkumab, a novel IL-23 inhibitor, might prove effective as alternatives for patients failing to improve on corticosteroids and anti-TNF treatment [137,138].

**Figure 1:**
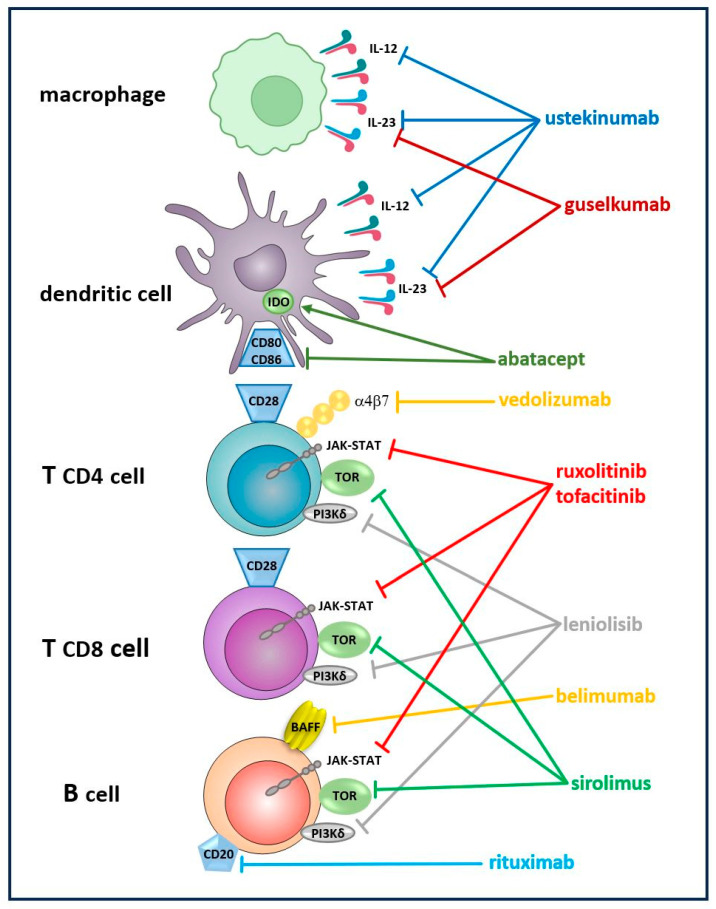
The molecular basis of the immunomodulatory therapies and their target cells. The inhibitory effect, → the stimulatory effect of the targeted immunomodulatory therapies, IDO indoleamine 2,3 dioxygenase.

The unveiled molecular basis for the disease opened a new perspective of targeted therapies, such as PI3Kδ inhibitor, leniolisib, modulating polyclonal proliferation and autoimmunity in PI3Kδ syndrome (APDS), or abatacept, a CTLA-4/FcIgG1 fusion protein effectively controlling T cell activation and autoimmune disorders in CTLA-4 haploinsufficiency or LRBA deficiency [140]. The JAK-STAT pathway inhibitors involving multiple proinflammatory cytokine signaling relevant for CVID may potentially lead to alleviation of inflammatory complications. The JAK inhibitors tofacitinib or ruxolitinib might be used in CVID enteropathy, psoriasis, and arthritis [137,141]. The molecular inhibitory and stimulatory effects of immunomodulatory therapies on target cells are displayed in Figure 1.

Uncovering the immunogenetic background for CVID and a better understanding of the molecular underpinnings for the organ-specific immunopathology pave the way for new patient-tailored therapies, improving the prognosis for affected patients. The progress of clustered regularly interspaced short palindromic repeats (CRISPR) gene editing technology revolutionized the clinical approach to biomedical sciences and clinical medicine, offering new perspectives for genetic disorders and new developments of precision medicine [142,143] (Table 1). Immunogenetic underpinnings of the organ-specific immunopathology relevant for CVID.

## Figures and Tables

**Table 1:** Immunogenetic underpinnings of the organ-specific immunopathology relevant for CVID.

Immune Dysregulation			
Genetic Variants	Immunophenotype	Organ-Specific Manifestation	Ref. No
*BACH 2* *BTB domain and CNC homolog 2*	Impaired B cell differentiation Defective immunoglobulin class-switch recombination and somatic hypermutation Excessive Th2 cell differentiation Impaired T reg cell development	Autoimmunity: vitiligo, endocrinopathies, IDDM, IBD	[120]
*CTLA-4* *Cytotoxic T lymphocyte antigen 4*	Low naïve T cells and recent thymic emigrants Low Treg cells	Autoimmunity: AIHA, ITP, enteropathy, IBD, endocrinopathies, arthritis, psoriasis Inflammatory granulomatosis: GLILD Asthma Lymphoproliferation: lymphadenopathy, splenomegaly	[121,122]
*ICOS* *Inducible T-cell costimulator*	Low B cell counts, in particular switched memory B cell and plasmablast deficiency Increased numbers of immature CD21low B cells	Autoimmunity: enteropathy, arthritis, IDDM	[123]
*IRF2BP2* *Interferon regulatory factor 2-binding protein 2*	Decreased B cell maturation, deficiency of switched memory B cells	Autoimmunity: IDDM, colitis, psoriasis	[124]
*LRBA* *Lipopolysaccharide (LPS)-responsive beige-like anchor protein*	Dysregulation of T cell activation and expansion Low B cell counts, in particular switched memory B cell and plasmablast deficiency Increased numbers of immature CD21low B cells	Autoimmunity: JIA, enteropathy, celiac disease, vitiligo, alopecia, AIHA, ITP, endocrinopathies, autoimmune hepatitis, IDDM Inflammatory: interstitial lung disease, bronchiectasis Inflammatory granulomatosis, GLILD Asthma Lymphoproliferation: lymphadenopathy, splenomegaly	[125,126,127]
*NFKB1* *nuclear factor kappa B1*	B cell lymphopenia, reduced non-switched and switched memory B cells CD21low B cell expansion	Autoimmunity: AIHA, ITP, vitiligo, alopecia, Hashimoto thyroiditis Lymphoproliferation: lymphadenopathy, splenomegaly Inflammatory granulomatosis: GLILD, granulomatous liver disease	[128]
*NFKB2* *Nuclear factor kappa B2*	Abnormalities in the T cell compartment: reduced numbers of follicular T helper cells, low Th17 cells, and Treg cells Impaired B cell differentiation: decreased switched memory and non-switched memory B cells, increased naïve B cells	Autoimmunity: arthritis, alopecia, vitiligo, ITP, growth hormone deficiency, ACTH deficiency, enteropathy Inflammatory: bronchiectasis Lymphoproliferation: splenomegaly, lymphadenopathy	[129]
*NOD2* *Nucleotide-binding oligomerization domain containing 2*	Impaired response to danger signals from pathogen-associated molecular patterns (PAMP) Deregulated inflammatory IL-6 homeostasis Disturbances in the actin cytoskeleton Disturbed B and T cell activation	Autoimmunity: IBD, IDDM, arthritis, multiple sclerosis, autoimmune encephalomyelitis Inflammatory granulomatosis Asthma	[130]
*STAT3* *Signal transducer and activator of transcription 3*	Disturbed regulation of TH17/Treg cell equilibrium B cell lymphopenia	Lymphoproliferation: lymphadenopathy, splenomegaly Autoimmunity: IDDM, growth hormone deficiency, Hashimoto thyroiditis, ITP, AIHA, enteropathy, arthritis Inflammatory: GLILD	[131]
*TACI* *Transmembrane activator and CAML interactor*	Impaired central and peripheral B cell tolerance BAFF driven B cell activation Treg cell dysfunction	Lymphoproliferation: splenomegaly, lymphadenopathy, tonsillar hypertrophy Autoimmunity: IDDM, JIA, ITP, AIHA, celiac disease, IBD Inflammatory granulomatosis	[132]

## Data Availability

The datasets generated during and/or analyzed during the current study are available from the corresponding author on reasonable request.
